# The inhibitory effects of a RANKL-binding peptide on articular and periarticular bone loss in a murine model of collagen-induced arthritis: a bone histomorphometric study

**DOI:** 10.1186/s13075-015-0753-8

**Published:** 2015-09-12

**Authors:** Genki Kato, Yasuhiro Shimizu, Yuki Arai, Natsuki Suzuki, Yasutaka Sugamori, Miki Maeda, Mariko Takahashi, Yukihiko Tamura, Noriyuki Wakabayashi, Ramachandran Murali, Takashi Ono, Keiichi Ohya, Setsuko Mise-Omata, Kazuhiro Aoki

**Affiliations:** Department of Pharmacology, Division of Bio-Matrix, Graduate School, Tokyo Medical and Dental University, Tokyo, 113-8549 Japan; Department of Orthodontic Science, Division of Oral Health Sciences, Graduate School, Tokyo Medical and Dental University, Tokyo, 113-8549 Japan; Department of Removable Partial Prosthodontics, Division of Oral Health Science, Graduate School, Tokyo Medical and Dental University, Tokyo, 113-8549 Japan; Present address: Department of Trauma-, Hand- and Reconstructive Surgery, Experimental Trauma Surgery, Molecular Skeletal Biology Laboratory, University Medical Center Hamburg-Eppendorf, Heisenberg Group, Martinistrasse 52, 20246 Hamburg, Germany; Department of Biomedical Sciences, Research Division of Immunology, Cedars-Sinai Medical Center, Los Angeles, CA 90048 USA

## Abstract

**Introduction:**

We designed OP3-4 (YCEIEFCYLIR), a cyclic peptide, to mimic the soluble osteoprotegerin (OPG), and was proven to bind to RANKL (receptor activator of NF-κB ligand), thereby inhibiting osteoclastogenesis. We recently found that another RANKL binding peptide, W9, could accelerate bone formation by affecting RANKL signaling in osteoblasts. We herein demonstrate the effects of OP3-4 on bone formation and bone loss in a murine model of rheumatoid arthritis.

**Methods:**

Twenty-four seven-week-old male DBA/1J mice were used to generate a murine model of collagen-induced arthritis (CIA). Then, vehicle or OP3-4 (9 mg/kg/day or 18 mg/kg/day) was subcutaneously infused using infusion pumps for three weeks beginning seven days after the second immunization. The arthritis score was assessed, and the mice were sacrificed on day 49. Thereafter, radiographic, histological and biochemical analyses were performed.

**Results:**

The OP3-4 treatment did not significantly inhibit the CIA-induced arthritis, but limited bone loss. Micro-CT images and quantitative measurements of the bone mineral density revealed that 18 mg/kg/day OP3-4 prevented the CIA-induced bone loss at both articular and periarticular sites of tibiae. As expected, OP3-4 significantly reduced the CIA-induced serum CTX levels, a marker of bone resorption. Interestingly, the bone histomorphometric analyses using undecalcified sections showed that OP3-4 prevented the CIA-induced reduction of bone formation-related parameters at the periarticular sites.

**Conclusion:**

The peptide that mimicked OPG prevented inflammatory bone loss by inhibiting bone resorption and stimulating bone formation. It could therefore be a useful template for the development of small molecule drugs for inflammatory bone loss.

**Electronic supplementary material:**

The online version of this article (doi:10.1186/s13075-015-0753-8) contains supplementary material, which is available to authorized users.

## Introduction

Many patients with rheumatoid arthritis (RA) still experience progressive bone erosion after primary treatment using disease-modifying antirheumatic drugs (DMARDs) [1]. Although the bone erosion of RA patients can be inhibited by tumor necrosis factor alpha (TNFα) neutralizing therapies [2], the direct inhibition of bone resorption has been proven to reduce the risk of bone fractures in patients with RA [3, 4]. The bone mineral density (BMD) of osteopenic patients is increased using bisphosphonates or an anti-receptor activator of nuclear factor-κB ligand (anti-RANKL) antibody, representative bone resorption inhibitors [5–7]; however, both of these treatments reduce the bone formation owing to their strong inhibition of bone resorption, leading to a low bone turnover rate [8]. Since the ratio of old bone to newly formed bone is increased by bone resorption inhibitors, the bone tends to accumulate microcracks, leading to a reduction of the bone quality, followed by atypical fractures [9–11]. In this context, a clinical trial using parathyroid hormone (PTH), a stimulator of bone formation and a bone resorption inhibitor [12], has been initiated to determine whether such treatment can reduce the fracture risk. Since RA is accompanied by a pathological state, which promotes bone resorption and reduces bone formation, an anabolic drug with anti-catabolic effects on bone is expected to be helpful to reduce the fracture risk.

OP3-4 (YCEIEFCYLIR) is a peptide that mimics osteoprotegerin (OPG). There are three binding sites for RANKL on OPG, and OP3-4 was designed based on the structure of the loop in the third cysteine-rich domain of OPG (OP3 site), one of the binding sites on OPG [13]. OP3-4 binds to RANKL with high affinity, thereby inhibiting osteoclastogenesis and bone resorption [13, 14]. Recently, the WP9QY peptide (W9), a different RANKL binding peptide derived based on the structures of tumor necrosis factor type 1 receptor/receptor activator of nuclear factor-κB (RANK) receptors [15, 16], was demonstrated to promote bone formation, as well as to inhibit bone resorption [17]. Since the ability of W9 to promote bone formation was decreased in osteoblasts isolated from RANKL-deficient mice calvariae or RANKL-downregulated osteoblasts (by small interfering RNA (siRNA) against RANKL), it was suggested that the stimulatory effects of RANKL binding peptides on bone formation might occur at least partially via a RANKL-dependent mechanism [17, 18]. We therefore hypothesized that the RANKL-binding peptide OP3-4 would also stimulate bone formation, as well as inhibit bone resorption.

In this study we performed osteoblast cultures to evaluate the direct effects of OP3-4 on osteoblast differentiation, and investigated the in-vivo effects of OP3-4 on bone resorption and bone formation using a murine model of collagen-induced arthritis (CIA). We herein demonstrate that OP3-4 promoted osteoblast differentiation and nodule formation in vitro. The peptide also prevented the increase in a serum bone resorption marker induced by CIA, and decreased the osteoclast number at the inflammatory sites. OP3-4 also prevented the CIA-induced bone loss at the periarticular sites of joints, promoting bone formation.

## Materials and methods

### Cell culture

Murine osteoclast precursors from 7-week-old male C57BL/6J mice were obtained from Nippon CLEA (Tokyo, Japan) and osteoclast-like cells were induced using cytokines, as described elsewhere [19]. In brief, murine bone marrow cells were cultured (6 × 10^5^ cells/well in a 48-well plate) in alpha-minimal essential medium (α-MEM; Sigma-Aldrich, St. Louis, MO, USA) containing 10 % fetal bovine serum (FBS; Invitrogen, Grand Island, NY, USA), and 100 U/ml penicillin, 100 μg/ml streptomycin (Sigma-Aldrich), and 25 ng/ml macrophage colony-stimulating factor (M-CSF; R&D Systems, Minneapolis, MN, USA) and 50 ng/ml RANKL (Wako, Osaka, Japan) in the presence or absence of either 0.05 % dimethyl sulfoxide (DMSO; Sigma-Aldrich), a control peptide (50 μM/l), or OP3-4 (1, 5, or 50 μM/l). The cells were incubated for 4 days at 37 °C. The OP3-4 peptide and the control peptide (FCYISEVEDQCY) were both purchased from American Peptide Company (Sunnyvale, CA, USA) [14]. Tartrate-resistant acid phosphatase (TRAP) staining was performed after fixation, and the number of TRAP-positive multinucleated cells (*n* >2) was counted.

An in-vitro osteoblastogenesis assay was carried out as described previously [19]. Briefly, primary osteoblast-like cells isolated from 1 day-old mice calvariae were seeded (5 × 10^4^ cells/well in a 24-well plate) and cultured in α-MEM (Sigma-Aldrich) with 10 % FBS (Hana-Nesco Bio, Brisbane, Australia) and 100 U/ml penicillin, 100 μg/ml streptomycin (Sigma-Aldrich) containing 50 mg/ml ascorbic acid (Wako), 10 mM β-glycerophosphate (Sigma-Aldrich), and 10 nM dexamethasone (Sigma-Aldrich). Alkaline phosphatase (ALP) staining was performed on day 7 and von Kossa staining was performed on day 21 of culture, and the ALP-positive and von Kossa-positive areas were measured using an image analysis system (KS400; CarlZeiss, Jena, Germany), as described previously [19].

### mRNA analyses

Primary osteoblast-like cells were cultured in the osteogenic medium as already described for the indicated days. Total RNA was isolated from culture in osteogenic medium using Trizol (Invitrogen) and was treated with DNase I (Invitrogen). cDNA was synthesized using PrimeScript II reverse transcriptase (Takara, Kyoto, Japan) according to the manufacturer’s instructions. Quantitative RT-PCR analysis was performed using SYBR Premix Ex Taq II (Takara) and a LightCycler 2.0 (Roche, Basel, Switzerland). For normalization, the expression of hypoxanthine guanine phosphoribosyl transferase (Hprt) was measured as an endogenous reference gene. The following primers specific for mouse were used: Hprt, sense 5′-CTT TGC TGA CCT GCT GGA TT-3′ and antisense 5′-TAT GTC CCC CGT TGA CTG AT-3′; Runx2, sense 5′-ACT GGC GGT GCA ACA AGA C-3′ and antisense 5′-CGG TAA CCA CAG TCC CAT CT-3′; Alp, sense 5′-GCA CCT GCC TTA CCA ACT CT-3′ and antisense 5′-TCA GGG CAT TTT TCA AGG TC-3′; and Bglap1/2 (ostocalcin), sense 5′-TAG TGA ACA GAC TCC GGC GCT ACC TT-3′ and antisense 5′-AGC TCG TCA CAA GCA GGG TTA AGC TC-3′. The relative levels of expression to cells maintained in α-MEM were calculated by the ΔΔCt method.

### Induction of CIA

The induction and assessment of CIA were performed as described previously [20]. Briefly, male DBA/1J mice (7 weeks old, six mice per group; Charles River Laboratories Japan, Kanagawa, Japan) were injected intradermally at the base of the tail with 200 μg bovine type II collagen (Collagen Research Center, Tokyo, Japan) in 0.05 M acetic acid (Sigma-Aldrich) emulsified in complete Freund adjuvant (CFA; Difco, Detroit, MI, USA). Twenty-one days after the primary immunization, the mice were boosted in the same way. The day of the first immunization was designated day 0. The mice were provided food (MF; Oriental Yeast Company, Tokyo, Japan) and distilled water ad libitum, and were maintained under a 12-hour light/dark cycle. The experimental procedures were reviewed and approved by the Animal Care and Use Committee of Tokyo Medical and Dental University (Tokyo, Japan) (authorization number: 120217A, 130255A, and 14070A).

### Treatment with the OP3-4 peptide

The OP3-4 peptide was dissolved in phosphate-buffered saline (PBS)-buffered 20 % DMSO (Sigma-Aldrich). Alzet osmotic minipumps (Model 2001 or 2002; Alza, Palo Alto, CA, USA) were prepared according to the manufacturer’s instructions. On day 28, the mice were anesthetized with injections of medetomidine hydrochloride (0.5 mg/kg; Meijiseika, Tokyo, Japan) and ketamine hydrochloride (50 mg/kg; Sankyo, Tokyo, Japan). A 1 cm incision was made in the skin, and the osmotic minipumps filled with 20 % DMSO (vehicle) or OP3-4 peptide (to deliver 9 mg/kg/day or 18 mg/kg/day) were subcutaneously implanted. The osmotic minipumps were replaced on day 35 and day 42, and infusions were continued until the mice were killed (on day 49). For measurement of the bone formation parameters, calcein (Sigma-Aldrich) was injected on days 42 and 47. The mice (six per group) were divided into four groups: nonimmunized mice receiving vehicle (20 % DMSO) (Normal-vehicle group); immunized mice receiving vehicle (20 % DMSO) (CIA-vehicle group); immunized mice receiving 9 mg/kg/day OP3-4 peptide (CIA-9 mg OP3-4 group); and immunized mice receiving 18 mg/kg/day OP3-4 peptide (CIA-18 mg OP3-4 group).

### Clinical assessment

To determine the arthritis score, two independent observers examined the mice daily from the day of the second immunization. The day of arthritis onset was considered to be when erythema and/or swelling was first observed. The severity of arthritis was graded on a 0–4 scale [21]. Briefly, the criteria for the grading were as follows: 0 = no evidence of erythema or swelling, 1 = erythema and mild swelling confined to the tarsals or ankle joint, 2 = erythema and mild swelling extending from the ankle to the tarsals, 3 = erythema and moderate swelling extending from the ankle to metatarsal joints, and 4 = erythema and severe swelling encompassing the ankle, foot, and digits, or ankylosis of the limb. Each paw was graded, and the four scores were added together so that the maximum possible score was 16 per mouse.

### Radiographic assessment of arthritis

At the end of the experiment (day 49), the mice were killed using domitor anesthesia, blood was collected from the orbital vein, and the hind paws were removed and fixed in phosphate-buffered glutaraldehyde (0.5 %)–formalin (4 %) fixative (pH 7.4) for 2 days, washed with PBS for 1 day and then used for the radiographic analyses. Three-dimensional reconstruction images and sagittal images of the knee joints were obtained by microfocal computed tomography (μCT) (Scan Xmate-E090; Comscan, Kanagawa, Japan) [22]. Microarchitectural changes were then measured using a three-dimensional bone structure analyzing system (TRI/3D-BON; RATOC System Engineering, Tokyo, Japan) [23]. To exclude the primary spongiosa, the region of interest (ROI) for the microstructural analyses of secondary spongiosa was set at 0.2–1.7 mm longitudinal length from the proximal end of epiphysis, and trabecular bones where bone separation was less than 60 μm (4 voxels) were excluded from the measurement [24]. For the analyses of epiphysis, the two-dimensional ROI (150 μm width) along the proximal end surface of tibiae was used. The BMD of the knee joints was measured by dual X-ray absorptiometry (DXA) (DCS-600R; Aloka, Tokyo, Japan) using the high-resolution scanning mode. The ROI (2.0 × 2.0 mm) for measuring the BMD at knee joints was determined by excluding the secondary spongiosa of the tibial and femoral metaphysis.

### Biochemical markers

The matrix metalloproteinase (MMP)-3, C-telopeptide fragments of type I collagen (CTX), and osteocalcin levels in the blood serum were analyzed according to the manufacturers’ instructions: for MMP-3, Mouse ELISA Kit (MMP 300) from R&D Systems, Inc. (Minneapolis, MN, USA); for CTX, RatLaps ELISA Kit (DS-AC 06F1) from Immunodiagnostic Systems Ltd (Fountain Hills, AZ, USA); and for osteocalcin, Mouse Gla-Osteocalcin High Sensitive EIA kit (MK 127) from Takara-Bio Inc. (Otsu-city, Shiga, Japan).

### Histological assessment of arthritis

The tibial bones were embedded in methyl methacrylate monomer (MMA), as described elsewhere [25]. In brief, polymerization was performed at 4 °C. Standard undecalcified sections (3 μm) were prepared using a fully automated rotary microtome Leica RM2265 device (Leica Biosystems, Nussloch GmbH, Germany). The direction of the cuts made in the bones was guided by μCT images of embedded samples before the sections were made. After removing MMA resin using 1-acetoxy-2-methoxyethane (Wako), the sections were then stained with TRAP and counterstained with toluidine blue. Histomorphometric analyses [25] were performed using the KS400 system (CarlZeiss). Some sections were stained according to the von Kossa method to detect calcified tissue, and were counterstained with modified van Gieson stain. Toluidine blue-stained sections were used to detect the pannus infiltration site at the articular cartilage of the tibial epiphysis.

### Histomorphometric evaluation of the epiphysis

The extent of cartilage degradation at the proximal end of the tibia was calculated using undecalcified toluidine blue-stained sections. The formula used was as follows:

$$ \mathrm{Extent}\ \mathrm{of}\ \mathrm{cartilage}\ \mathrm{degradation}\ \left(\%\right) = \left(\mathrm{length}\ \mathrm{of}\ \mathrm{cartilage}\ \mathrm{degradation}\right)\ /\ \left(\mathrm{total}\ \mathrm{length}\ \mathrm{of}\ \mathrm{proximal}\ \mathrm{end}\ \mathrm{of}\ \mathrm{tibial}\ \mathrm{epiphysis},\ \mathrm{excluding}\ \mathrm{anterior}\ \mathrm{intercondylar}\ \mathrm{area}\right) \times 100. $$

The undecalcified sections that were stained with TRAP and counterstained with toluidine blue were used for the assessments. The calcified area/tissue area and osteoclast number/bone volume (N.Oc/BV) ratios in the epiphysis of the proximal tibia were measured using the KS400 image analyzing system, as described previously [20]. TRAP-positive multinucleated (*n* >2) cells that formed resorption lacunae on the surface of the trabeculae were designated osteoclasts. The labeled surface was also measured in the region of epiphyses.

### Bone histomorphometry of the tibial metaphysis

To investigate the secondary effects of CIA on bone resorption, in addition to the resorption of periarticular bone, standard histomorphometric analysis in the tibial metaphysis was performed [20, 26] using the image analysis system already described.

The ROI for the histomorphometric analyses of the metaphysis was set at 0.6 mm distal from the center of the growth plate and the size of the ROI was 1.1 mm (longitudinal length) × 0.7 mm (width) at the center of diaphysis to exclude the primary spongiosa.

### Statistical analysis

The Kruskal–Wallis test was performed to analyze the arthritis score. For comparison purposes of each group, the Mann–Whitney U test with Bonferroni correction was applied. The other data were analyzed by an analysis of variance. When an *F* test yielded significant results (*p* <0.05), the groups were compared using Fisher’s protected least significant difference post-hoc test. Tests were carried out using an Apple software program, StatView 4.1 (SAS Institute, Cary, NC, USA). Values of *p* <0.05 were considered significant.

## Results

### The effects of OP3-4 on osteoclastogenesis and osteoblast differentiation in vitro

**Fig. 1:**
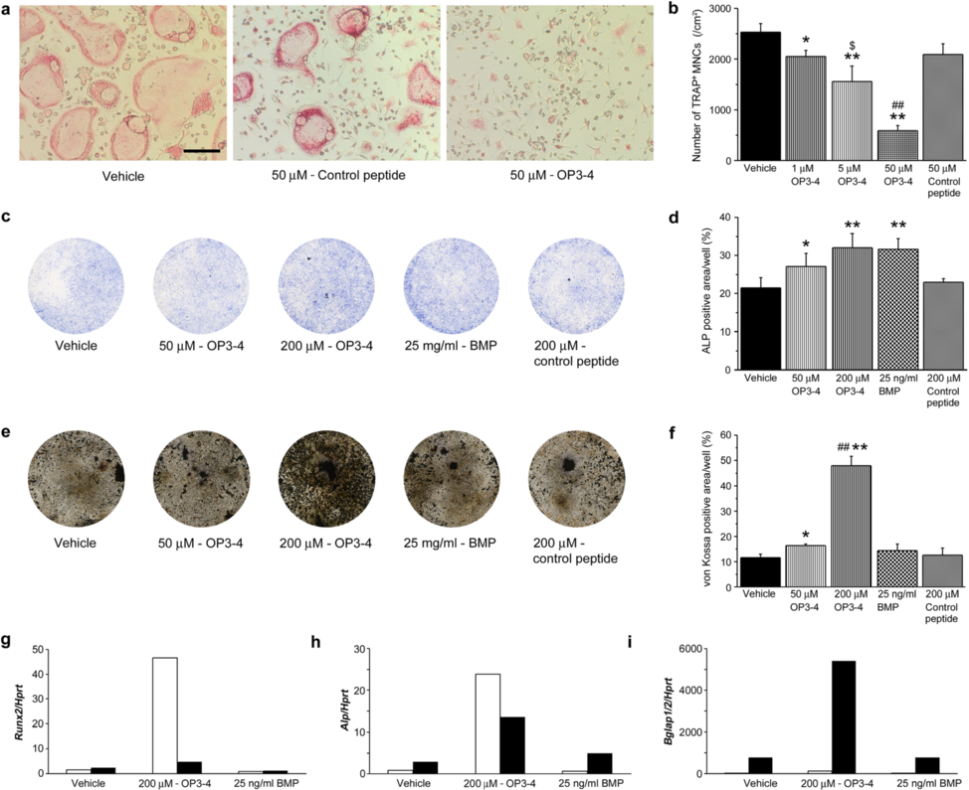
Direct effects of OP3-4 on osteoclastogenesis and osteoblast differentiation. **a** Microscopic view of osteoclast formation in vitro. TRAP-positive cells are shown (*red*). Bone marrow macrophages were treated with 25 ng/ml M-CSF and 50 ng/ml RANKL for 4 days. Scale bar represents 100 μm. **b** Number of TRAP-positive multinucleated cells. Data presented as mean ± standard deviation (*SD*). *p <0.05, **p <0.01 vs. vehicle control, ^$^ *p* <0.05 vs. 1 μM OP3-4, ^##^ *p* <0.01 vs. 5 μM OP3-4. **c** ALP-positive cells in the cultures of primary osteoblastic cells on day 7. **d** Percentage of ALP-stained areas in each well. Data presented as mean ± SD. *p <0.05, **p <0.01 vs. vehicle control. **e** von Kossa-positive cells in the cultures of primary osteoblastic cells on day 21. **f** Percentage of von Kossa-stained areas in each well. Data presented as mean ± SD. *p <0.05, **p <0.01 vs. vehicle control; ^##^ *p* <0.01 vs. 50 μM OP3-4. All cultures were performed at least three times independently, and similar data were obtained each time. Representative images of cultures and quantitative data are provided (*n* = 3–4/group in each independent experiment). **g–i** Gene expression analyses of *Runx2* **g**, *Alp* **h**, and *Bglap1/2* **i** by quantitative PCR were performed using the cells on day 5 (*white bar*) and day 14 (*black bar*). *BMP* bone morphogenetic protein

Because OP3-4, an OPG mimetic peptide, is known to inhibit osteoclastogenesis [13, 14], we first tried to confirm its effects on osteoclast formation in vitro. Bone marrow cells from tibiae and femurs were cultured in the presence of soluble RANKL and M-CSF for 4 days, and the TRAP-positive multinucleated cells were counted. As shown in Fig. 1a, b, OP3-4 inhibited the formation of TRAP-positive multinucleated cells in a concentration-dependent manner. The 50 μM concentration of OP3-4 decreased the number of TRAP-positive cells by 76 % (Fig. 1b). On the contrary, 50 μM control peptide had little effect on osteoclast-like cell formation.

Next, primary osteoblast-like cells were isolated from 1-day-old mice calvariae, and were cultured in the presence or absence of OP3-4 for 7 or 21 days. As shown in Fig. 1c, d, the number of ALP-positive cells on day 7 of culture was found to be significantly increased in a concentration-dependent manner by OP3-4. The bone nodule formation was determined by von Kossa staining on day 21. As shown in Fig. 1e, f, the von Kossa-positive area was significantly increased in a concentration-dependent manner by OP3-4. In parallel with the results, OP3-4 markedly enhanced mRNA expression of *Runx2*, *Alp*, and *Bglap1/2* (genes of osteocalcin), as shown in Fig. 1g–i, suggesting that OP3-4 enhances osteoblast differentiation as well as inhibiting osteoclastogenesis in vitro*.*

### OP3-4 treatment did not reduce the inflammatory indices

**Fig. 2:**
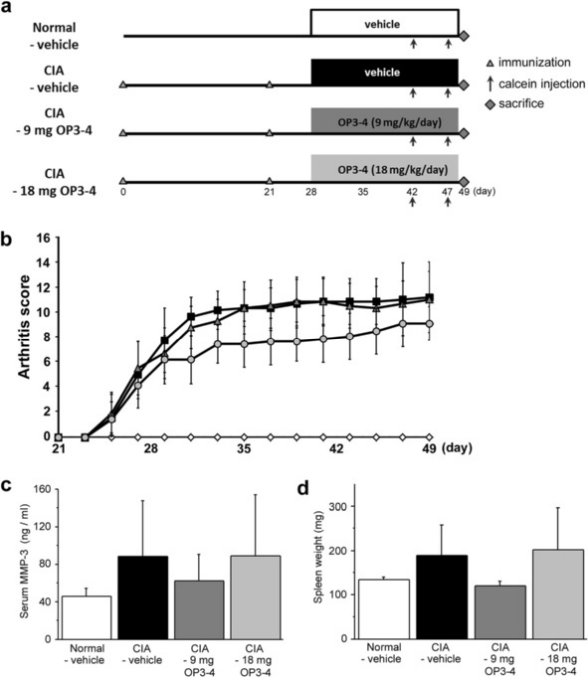
The in-vivo experimental protocol, the clinical score, and the inflammatory indices. **a** Collagen-induced arthritis (*CIA*) was induced by primary (day 0) and secondary (day 21) immunizations with bovine type II collagen in CFA. Infusion pumps were implanted subcutaneously in mice with CIA on day 28. **b** Arthritis scores (clinical severity of arthritis). The maximum possible score is 16, as described in Materials and methods. Shown are the results of vehicle-treated mice with CIA (*closed squares*), mice with CIA treated with 9 mg/kg/day OP3-4 peptide (*closed triangles*), mice with CIA treated with 18 mg/kg/day OP3-4 peptide (*closed circles*), and vehicle-treated nonimmunized mice (*open diamond*). Values are mean ± SD (*n* = 6 mice per group). **c** Serum matrix metalloproteinase (MMP)-3 levels on day 49. **d** The spleen weight was measured on day 49. Normal-vehicle group, nonimmunized mice receiving vehicle (20 % DMSO); CIA-vehicle group, immunized mice receiving vehicle (20 % DMSO); CIA-9 mg OP3-4 group, immunized mice receiving 9 mg/kg/day OP3-4 peptide; CIA-18 mg OP3-4 group, immunized mice receiving 18 mg/kg/day OP3-4 peptide

To clarify the effects of OP3-4 peptide on inflammatory bone destruction, we evaluated its in-vivo effects in a murine model of CIA (Fig. 2a). First, we assessed the development of inflammation by scoring the clinical disease activity daily from day 21 after the first immunization, as described in Materials and methods. The disease activity in vehicle-treated mice with CIA first appeared on day 25, which was 4 days after the second immunization (Fig. 2b). Vehicle or OP3-4 administrations were started after the onset of the disease (day 28). The grade of arthritis was not reduced significantly by the OP3-4 treatments during the experimental period, although treatment with the higher dose of OP3-4 tended to reduce the arthritis score (Fig. 2b). When we measured the serum levels of MMP-3, an inflammatory marker, at the end of the experiment, the levels were not increased in the CIA-vehicle group, and no significant differences were detected among the experimental groups (Fig. 2c). In addition, the changes in spleen weight were comparable with those of the serum MMP-3 levels (Fig. 2d).

When we administered vehicle or OP3-4 from the same day as the first immunization, OP3-4 did not affect significantly the grade of arthritis, such as reduction of body weight, arthritis score, and swelling of paws (Fig. S1A–C in Additional file 1). Histological observation around the hind limb heels (calcaneus) revealed infiltration of the mononuclear cells into the joints in both vehicle-treated and OP3-4-treated mice (Fig. S1E in Additional file 1). We did not detect a significant difference in the inflammatory score between the two groups (Fig. S1F in Additional file 1). We also scored the number of proliferating cells in the periarticular regions of calcaneus, which were positive for Ki-67 antigen by immunohistochemical staining, but we did not detect a difference between vehicle-treated and OP3-4-treated mice (Fig. S1G in Additional file 1). These observations suggest that OP3-4 had a limited effect on CIA progression and inflammatory responders.

### OP3-4 treatment inhibited CIA-induced BMD as measured by the μCT and DXA analyses

**Fig. 3:**
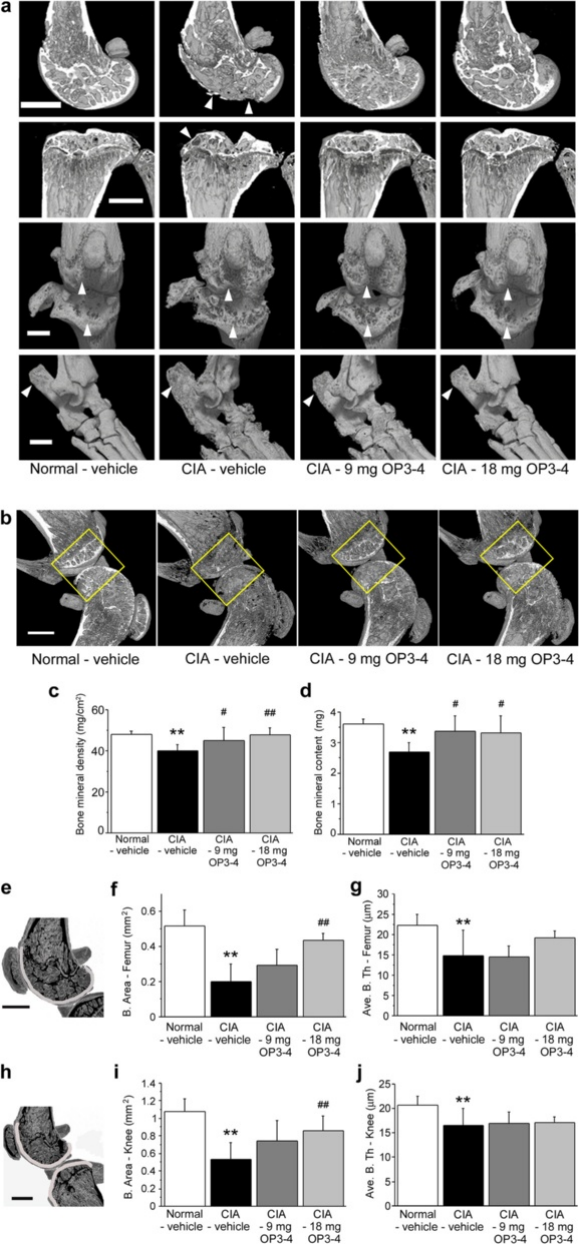
Radiographic observations of femurs, tibiae, knee joints, and ankle joints. **a** μCT was used to clarify the structural changes of bones. *Arrowheads*, erosion surface or site of the destruction of bones. OP3-4 appeared to inhibit the bone loss induced by collagen-induced arthritis (*CIA*). Scale bar represents 1 mm. **b** ROIs used for the quantitative measurements of mineralized tissue at knee joints by DXA. The square areas (2 × 2 mm) are the ROIs used for the DXA analyses. **c** BMD and **d** bone mineral content at the joint. The regions of interest shown in light gray for measuring the indices (f/g and i/j) to show the changes of e femur epiphysis and h knee joint, respectively. Data presented as mean ± SD (*n* = 6). **p <0.01 vs. Normal-vehicle; ^#^ *p* <0.05, ^##^ *p* <0.01 vs. CIA-vehicle. Normal-vehicle group, nonimmunized mice receiving vehicle (20 % DMSO); CIA-vehicle group, immunized mice receiving vehicle (20 % DMSO); CIA-9 mg OP3-4 group, immunized mice receiving 9 mg/kg/day OP3-4 peptide; CIA-18 mg OP3-4 group, immunized mice receiving 18 mg/kg/day OP3-4 peptide

When we observed the three-dimensional μCT reconstruction images of femurs, tibiae, knee joints, and ankle joints, the CIA-vehicle group showed a reduction of the radio-opaque area and bone erosion surface compared with the Normal-vehicle group (Fig. 3a), confirming the validity of the model. The OP3-4 treatment groups exhibited recovery from the CIA-induced reduction of the radio-opaque area and bone erosion surface (Fig. 3a, b).

To confirm the μCT observations, quantitative DXA analyses were performed. Figure 3b shows the ROIs at the knee joints for the DXA analyses. OP3-4 treatment significantly inhibited the CIA-induced reduction of the BMD and the bone mineral content, in a dose-dependent manner (Fig. 3c, d). The reduction of BMD was also inhibited when OP3-4 was administered from the first immunization time point (Fig. S1D in Additional file 1). We also performed two-dimensional analyses of the bone area close to the knee joint space, shown in Fig. 3e, h. The significant reduction of bone area and average bone thickness were observed in the vehicle-administered CIA group compared with the Normal-vehicle group, but the 18 mg/kg/day OP3-4 administration prevented the reduction significantly (Fig. 3e–j).

### OP3-4 prevented the articular cartilage destruction and subchondral bone destruction at the epiphysis in the CIA model

**Fig. 4:**
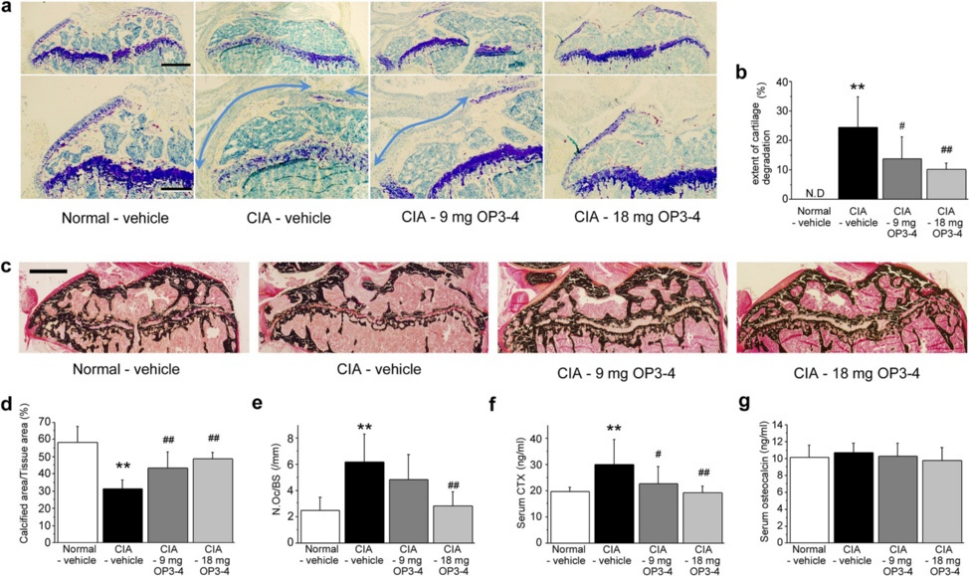
Histological observations and quantitative analyses of the articular sites of tibiae. **a** Representative microscopic images of toluidine blue-stained undecalcified sections. The area of metachromasia after toluidine blue staining was decreased in the CIA-vehicle group. *Arrows*, length of the cartilage degradation site facing the proximal end of the tibiae. Scale bars represent 500 μm and 250 μm in the upper and lower panels, respectively. **b** The extent of cartilage degradation = (length of cartilage degradation surface) / (total length of proximal end of tibial epiphysis, excluding anterior intercondylar area) × 100. **c** von Kossa-stained sections at the tibial epiphysis. **d** Calcified area measurements at the tibial epiphysis obtained by bone histomorphometry. Scale bar represents 1 mm. **e** Osteoclast number per bone surface (*N.Oc/BS*) at the tibial epiphysis. **f** Serum levels of C-terminal telopeptides of type I collagen (*CTX-I*) on day 49. **g** Serum levels of osteocalcin on day 49. Data presented as mean ± SD (*n* = 6). **p <0.01 vs. Normal-vehicle; ^#^ *p* <0.05, ^##^ *p* <0.01 vs. CIA-vehicle. Normal-vehicle group, nonimmunized mice receiving vehicle (20 % DMSO); CIA-vehicle group, immunized mice receiving vehicle (20 % DMSO); CIA-9 mg OP3-4 group, immunized mice receiving 9 mg/kg/day OP3-4 peptide; CIA-18 mg OP3-4 group, immunized mice receiving 18 mg/kg/day OP3-4 peptide. *CIA* collagen-induced arthritis

Histological observations of the toluidine blue-stained sections are shown in Fig. 4a. The area of metachromasia indicated by toluidine blue staining at the articular site of tibiae was reduced in the CIA-vehicle group compared with the Normal-vehicle group. When compared with the CIA-vehicle group, OP3-4 seemed to increase the area of metachromasia at the articular site of tibiae. To clarify the effects of OP3-4 on cartilage destruction, we measured the articular surface of proximal tibiae, which had no area of metachromasia following toluidine blue staining. To clarify the cartilage degradation, we designated the cartilage degradation surfaces as “an extent of cartilage degradation”. When the length ratio of the “cartilage degradation surface” to the total length of the proximal end of the tibial epiphysis was calculated, the CIA-vehicle group showed a significantly increased value compared with the Normal-vehicle group, in which the “cartilage degradation surface” was not detectable (Fig. 4b). This histomorphometric assessment revealed that OP3-4 treatment significantly inhibited CIA-induced cartilage degradation in a dose-dependent manner (Fig. 4b). The enhancement of proliferation and differentiation of cartilage cell line by the OP3-4 treatment supported these observations in vivo (Additional file 2).

To assess the effects of OP3-4 on articular bone destruction and bone metabolism, a histomorphometric analysis was also performed on the tibial epiphysis. OP3-4 seemed to prevent the CIA-induced reduction of the trabecular thickness (Fig. 4c). Quantitatively, the CIA-vehicle group exhibited a significantly decreased calcified area compared with the Normal-vehicle group. Both OP3-4 treatment groups had greater calcified areas compared with the CIA-vehicle group (Fig. 4d). The number of osteoclasts per bone surface (N.Oc/BS) was significantly increased in the CIA-vehicle mice compared with Normal-vehicle mice. This higher ratio in N.Oc/BS was significantly reduced in both OP3-4-treated groups (Fig. 4e). These results indicate that OP3-4 peptide could inhibit the CIA-induced bone loss and bone erosion at the tibial epiphysis. To clarify the systemic bone resorption activity, serum CTX levels were measured and were found to be significantly greater in the CIA-vehicle group compared with the Normal-vehicle group. This high level of serum CTX was also significantly reduced in both OP3-4-treated groups (Fig. 4f). The serum levels of osteocalcin, a bone formation marker, were similar in all four groups at the end of the experiments (day 49) (Fig. 4g).

### OP3-4 prevented periarticular bone loss at the tibial metaphysis in CIA mice

**Fig. 5:**
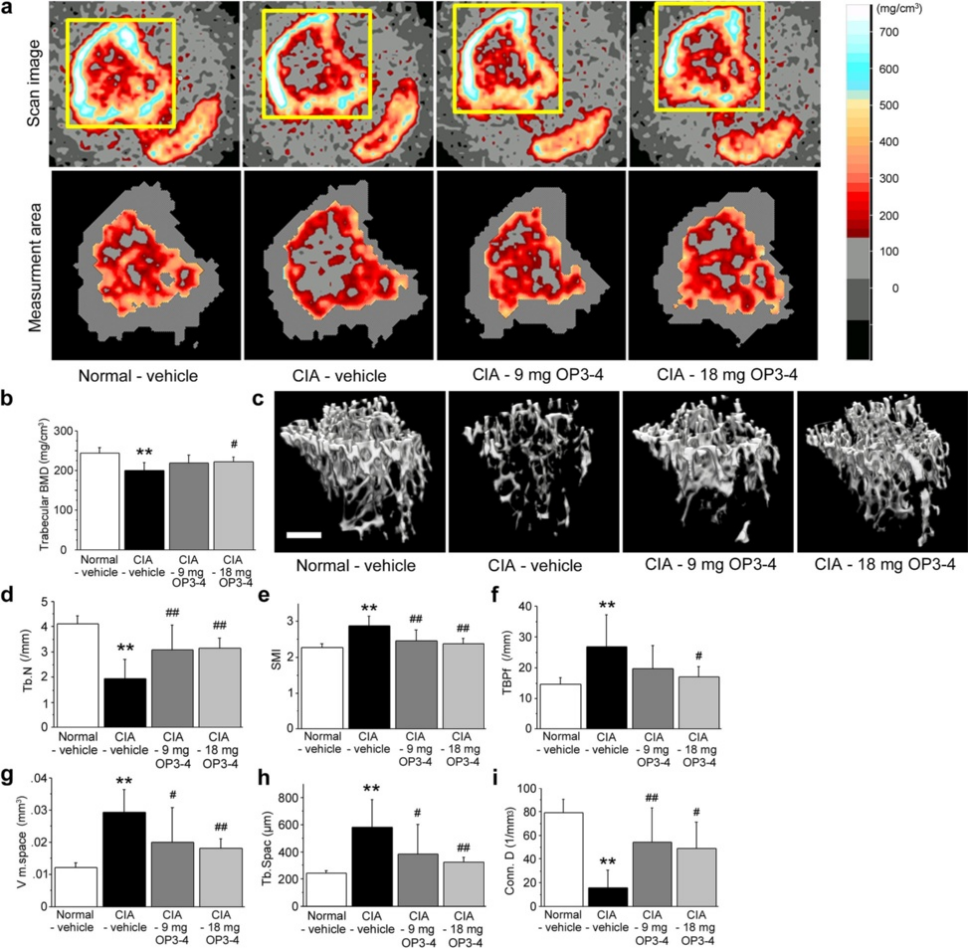
OP3-4 prevented CIA-induced peripheral bone loss at the tibial metaphysis. **a, b** pQCT was used to measure the bone mineral density (*BMD*) at the secondary spongiosa of the tibiae. *Upper panels*, representative scan images from the pQCT analyses; *lower panels*, trabecular area for each group; *right panel*, color table for the BMD. **c** μCT reconstruction images of the tibial metaphysis. Scale bar represents 200 μm. **d** Trabecular number (*Tb.N*; measure of the average number of trabeculae per unit length). **e** Structure model index (*SMI*; an indicator of the structure of trabeculae). SMI will be 0 for parallel plates and 3 for cylindrical rods. **f** Trabecular bone pattern factor (*TBPf*; an indicator of morphological changes of trabecular surface). **g** Marrow space star volume (*V m. space*; an indicator of osteoporotic changes). **h** Trabecular spacing (*Tb.Spac*). **i** Degree of connectivity of trabeculae normalized by tissue volume (*Conn.D*). Data expressed as mean ± SD (*n* = 6) for each group. **p <0.01 vs. Normal-vehicle; ^#^ *p* <0.05, ^##^ *p* <0.01 vs. CIA-vehicle. Normal-vehicle group, nonimmunized mice receiving vehicle (20 % DMSO); CIA-vehicle group, immunized mice receiving vehicle (20 % DMSO); CIA-9 mg OP3-4 group, immunized mice receiving 9 mg/kg/day OP3-4 peptide; CIA-18 mg OP3-4 group, immunized mice receiving 18 mg/kg/day OP3-4 peptide. *CIA* collagen-induced arthritis

Since it is well known that CIA causes periarticular bone loss, as well as articular bone destruction, the secondary spongiosa in the tibial metaphysis was also analyzed using peripheral quantitative computed tomography (pQCT) and μCT reconstruction images. Figure 5b shows the ROIs for BMD measurements of the tibial secondary spongiosa by pQCT, indicating that the longitudinal positioning of the ROI setting was correct, since the distance between the fibula and tibia was almost the same among the different groups (Fig. 5a, upper panel).

The trabecular BMD of the tibial metaphysis was reduced in the CIA mice compared with the Normal-vehicle group (Fig. 5b). Treatment with the 18 mg/kg/day OP3-4 infusion significantly inhibited the decrease in the trabecular BMD induced by CIA (Fig. 5b).

The μCT images showed the micro-architectural changes of the tibial metaphysis. The CIA-vehicle group showed a reduction in the trabecular number (Tb.N; Fig. 5d) and the degree of connectivity of trabeculae normalized by tissue volume (Conn.D; Fig. 5i), and a high level of the architectural indices structure model index (SMI; Fig. 5e), trabecular bone pattern factor (TBPf; Fig. 5f), marrow space star volume (V m.space; Fig. 5g), and trabecular spacing (Tb.Spac; Fig. 5h), while OP3-4 treatments significantly prevented the decrease in Tb.N and Conn.D induced by CIA and the increase in the SMI, TBPf, V m.space, and Tb.Spac (Fig. 5d–i).

### OP3-4 promoted the bone formation parameters in the secondary spongiosa at the tibial metaphysis in CIA mice

**Fig. 6:**
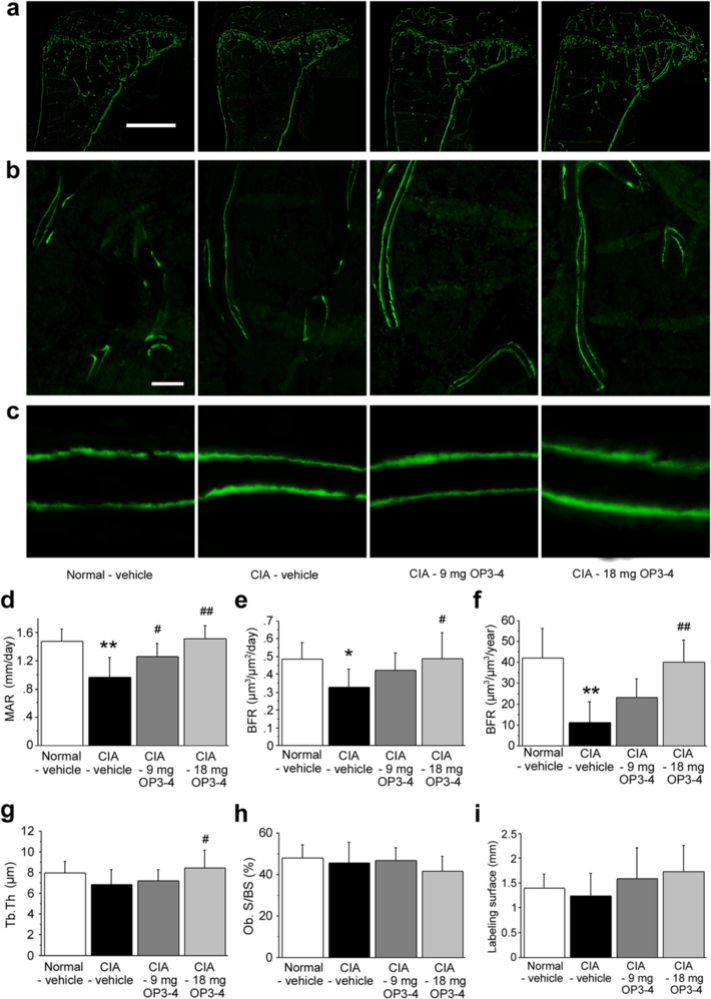
Fluorescent images of undecalcified sections of the periarticular sites in tibiae. **a** Representative fluorescent images of the proximal tibiae. Scale bar represents 1 mm. **b** Representative fluorescent images of the secondary spongiosa of the tibiae. Scale bar represents 50 μm. **c** Magnified images of calcein double labeling of the trabecular bone. **d** Mineral apposition rate (*MAR*)/day. **e** Bone formation rate (*BFR*)/day in a bone surface reference. **f** BFR/year in a total tissue reference. **g** Trabecular thickness (*Tb.Th*). **h** Osteoblast surface per bone surface (*Ob.S/BS*). **i** Labeling surface at epiphysis. Data expressed as mean ± SD (*n* = 6) for each group. *p <0.05, **p <0.01 vs. Normal-vehicle; ^#^ *p* <0.05, ^##^ *p* <0.01 vs. CIA-vehicle. Normal-vehicle group, nonimmunized mice receiving vehicle (20 % DMSO); CIA-vehicle group, immunized mice receiving vehicle (20 % DMSO); CIA-9 mg OP3-4 group, immunized mice receiving 9 mg/kg/day OP3-4 peptide; CIA-18 mg OP3-4 group, immunized mice receiving 18 mg/kg/day OP3-4 peptide. *CIA* collagen-induced arthritis

To clarify the effects of OP3-4 on the bone formation in the CIA model, calcein injections were performed 7 days and 2 days before sacrifice. When we observed undecalcified thin sections under a fluorescent microscope, the distance between the calcein double labels seemed to be narrower in the CIA-vehicle group than in the Normal-vehicle group, and OP3-4 seemed to prevent the CIA-induced reduction of the distance (Fig. 6a–c). To confirm these histological observations, a bone histomorphometric study was performed. The mineral apposition rate (MAR), which indicates the bone formation ability of osteoblasts, was significant reduced in the CIA-vehicle group compared with the Normal-vehicle group, while OP3-4 treatment prevented the reduction of the MAR in a dose-dependent manner (Fig. 6d). The mineralizing surface per bone surface (MS/BS) was not significantly different among the four groups (data not shown). Consequently, the bone formation rate (BFR), which reflects the total amount of bone formation in a day, showed similar changes to those of the MAR since BFR is defined as follows; BFR = MS/BS × MAR (Fig. 6e). The BFR in a total tissue reference, which shows the total amount of calcified tissue in a year, also showed similar or even prominent changes compared with the BFR in a bone surface reference (Fig. 6f). The trabecular thickness and the osteoblast surface were also measured (Fig. 6g, h). To clarify the site difference in bone formation activity, we measured the labeling surface at the tibial epiphysis, but no significant effects appeared among groups (Fig. 6i).

## Discussion

In this study we first demonstrated the concentration-dependent acceleration of osteoblast differentiation and nodule formation by OP3-4, an OPG mimetic, in vitro. Secondly, in the murine CIA model, although OP3-4 did not significantly ameliorate the CIA symptoms the peptide did increase the MAR, and consequently the BFR, at the tibial secondary spongiosa, which was clearly demonstrated by a histomorphometric study using calcein double labeling. Since the MAR indicates the function of osteoblasts in terms of the calcification, our data suggest that OP3-4 also promoted bone formation in vivo. We also confirmed the inhibitory effects of OP3-4 on osteoclastogenesis. These changes in the bone at periarticular sites were similar to those induced by OPG treatment, as reported previously [27], but the mechanism by which the treatment prevents bone loss may be different, since OPG does not have an anabolic effect on bone. Taken together, our study suggests that subcutaneous injections of OP3-4 prevented the periarticular bone loss at the secondary spongiosa of the tibial metaphysis, inhibiting bone resorption and promoting bone formation.

One of the stimulatory effects of OP3-4 on bone formation is thought to be exerted via a reversal in the signaling of membrane-bound RANKL, similar to the findings of our previous study, where we proposed that another RANKL-binding peptide (W9) could promote bone formation through membrane-bound RANKL in osteoblasts [17]. Although the equilibrium constant (Kd) values of W9 and OP3-4 for RANKL are 3.76 × 10^−7^ [28] and 3.89 × 10^−6^ [13], respectively, the binding dissociation constant (Koff), an important parameter of the affinity, is comparable between the two molecules (W9, 2.85 × 10^−3^; OP3-4, 9.37 × 10^−4^ [13]), suggesting that both W9 and OP3-4 similarly bind to RANKL, making a stable complex, then lead to the reversal signaling in osteoblasts. Since PTH can work as an anabolic peptide only when osteoclasts are present, some factors derived from osteoclasts are thought to be necessary for the anabolic effects of PTH [29, 30]. In contrast, OP3-4 might directly stimulate osteoblasts via a mechanism other than that involving PTH.

The RANKL reverse-signaling phenomena have already been clarified in an osteoblastic cell line with regard to RANKL membrane trafficking [31]. Kariya et al. [31] demonstrated that the interaction between RANK-Fc-conjugated beads (RANK-coated beads) and membrane-bound RANKL leads to an increase in membrane-bound RANKL by promoting the trafficking of RANKL-containing vesicles to the cell membrane. RANK-coated beads also stimulate ALP activity [32]. RANK-Fc alone does not stimulate ALP activity, but the oligomerization of RANK-Fc by IgM or RANK-Fc conjugation on the surface of beads can stimulate ALP activity [32]. The OP3-4 peptide, which works as a small RANK [13], stimulated ALP activity only at the higher concentration (200 μM). At higher concentrations, the OP3-4 peptide seemed to be precipitated in vitro, suggesting aggregation of the peptide at higher concentration which might induce conformational changes that resemble RANK oligomerization, leading to RANKL clustering on the surface of osteoblasts. The promotion of RANKL trafficking could help to induce RANKL clustering on the cell membrane. The RANKL-binding peptide could stimulate bone formation, but neither OPG nor an anti-RANKL antibody could stimulate bone formation [33, 34]. This might be because OPG and the anti-RANKL antibody cannot induce auto-oligomerization [35]. The higher concentration of the RANKL-binding peptide could stimulate the auto-oligomerization and induce RANKL clustering, which might switch on the RANKL-reverse signaling to stimulate ALP activity. Similar inverse-agonistic activity by CD4 peptide mimics in T-cell activation has been observed [36]. Further studies are necessary to clarify the detailed mechanism(s) underlying the effects of OP3-4 on bone formation.

In this study, OP3-4 treatment seemed to reduce the CIA-induced clinical score, although not significantly. The high dose of OP3-4 may seem to reduce the clinical score because of secondary effects resulting from the prevention of CIA-induced bone loss. Because a score of 4 was defined as ankylosis of the limb, the strong inhibitory effect of OP3-4 on bone loss may have prevented the development of ankylosis of the limb, leading to a reduction of the clinical score in the high-dose OP3-4-treated mice. Conversely, OP3-4 may not prevent the induction of immune response nor inflammatory response. Infiltration of mononuclear cells into the joints and proliferation of cells in periarticular region were not inhibited even in mice administered OP3-4 immediately before the first immunization. We did not see an increase in the serum MMP-3 level, an inflammatory marker, in the CIA group, suggesting that the mice had already passed the peak of inflammation at the time of sacrifice. In addition, the serum osteocalcin level was not reflected in the data for the bone formation parameters at the secondary spongiosa of the tibia, as shown in Fig. 6, also suggesting that the mice in the CIA group had passed the peak reduction in bone formation by the time of sacrifice.

The discrepancy between the serum osteocalcin level and bone formation parameters at the periarticular site could be explained by the restricted changes at the secondary spongiosa. The region we measured as a periarticular site was composed of a small bone volume compared with the whole skeletal bone, including the cortical bone compartment. This could be the reason why the bone histomorphometric changes of the bone formation indices at the periarticular site did not reflect the changes of the serum osteocalcin levels or the systemic changes of bone formation.

In this study, OP3-4 prevented the CIA-induced cartilage destruction. Since the major cartilage destruction and subchondral bone destruction are thought to be mediated by osteoclasts existing at the leading edge of the pannus [37, 38], the inhibitory effects of OP3-4 on osteoclasts are thought to prevent the cartilage destruction, as alendronate treatment was reported to prevent cartilage destruction in a rabbit model of osteoarthritis [39]. On the other hand, the injection of denosumab, an anti-RANKL antibody, was reported to have a much smaller effect on joint space narrowing and cartilage destruction compared with TNFα neutralizing therapy, since it does not affect joint inflammation [40]. These reports suggest that the inhibition of cartilage destruction by OP3-4 might not be due merely to the inhibition of osteoclasts. Furthermore, the effects of OPG on cartilage destruction are varied, although there is consensus that OPG does not affect inflammation [27, 41, 42]. In our preliminary experiments, we found the OP3-4 could stimulate the proliferation and differentiation of ATDC5 mouse embryonic chondrocyte cells (Fig. S2 in Additional file 2). Further studies will also be necessary to clarify the mechanism by which OP3-4 inhibits cartilage destruction.

When comparing macromolecules, the peptide-type drug candidate OP3-4 might have fewer side effects and lower production costs than agents such as antibodies [43]. However, with regard to clinical applications, a sustained-release carrier for OP3-4 needs to be developed since peptide drugs are unstable and aggregate easily in vivo*.* The effects of OP3-4 in this study were achieved using infusion pumps subcutaneously implanted into the backs of the mice. As we have previously shown, a cholesterol-bearing pullulan (CHP) can be used to make nanogels, or gelatin hydrogels could be candidate peptide carriers [44, 45]. In the case of the W9 peptide, the use of CHP nanogels could reduce the injection frequency from eight times a day to once or twice a day (subcutaneous injections) while still maintaining the inhibitory effects of W9 on bone resorption [45, 46]. For the systemic administration of peptide drug, the development of carriers that can prevent peptide aggregation and allow the peptide drug to remain biologically functional is essential prior to clinical use.

## Conclusion

We have herein demonstrated that the RANKL-binding peptide OP3-4 increased bone formation at periarticular sites, and also decreased bone resorption, preventing bone loss due to CIA. The peptide drug, which was designed based on OPG, could be a lead drug candidate for RA treatment, and both inhibits bone resorption and stimulates bone formation, leading to less bone loss and a higher quality of the bones.

## Additional files

Additional file 1: Figure S1. Showing that OP3-4 administration has a limited effect on the induction and inflammation of arthritis. **A–C** The osmotic minipumps filled with 20 % DMSO (vehicle) or OP3-4 peptide (to deliver 18 mg/kg/day) were implanted subcutaneously before the first immunization. Body weight **A**, arthritis score **B**, and paw swelling by caliper measurement **C** of mice with CIA treated with vehicle (*closed squares*), mice with CIA treated with 18 mg/kg/day OP3-4 peptide (*closed circles*), and normal mice treated with vehicle (*open diamond*) are indicated. There is no significant difference between vehicle-treated and OP3-4-treated mice. **D** BMD measured by DXA. ***p* <0.01 vs. Normal-vehicle, ^#^ *p* <0.05 vs. CIA-vehicle. **E** Histological images of hematoxylin and eosin staining of hind limb heels are shown. *Lower panels*, higher magnification of the squares in the upper panels. Bars indicate 1 mm. **F** Inflammatory scores in each group of mice show no significant difference between CIA-vehicle and CIA-OP3-4 mice. ***p* <0.01 vs. Normal-vehicle. **G** Numbers of proliferating cells which are positive for Ki-67 antigen counted in the periarticular region. No significant differences are detected. (JPEG 3012 kb) Additional file 2: Figure S2. Showing the effects of OP3-4 on the proliferation and differentiation of cartilage cell line ATDC5. **A** Results of proliferation assay on day 1 with the noninduction medium. **B** Alcian blue-positive area ratio in the cartilage induction medium on day 10. ***p* <0.01 vs. vehicle control, #*p* <0.05 vs. 100 μM OP3-4. (JPEG 384 kb) Additional file 3:
**Presents supplementary methods.** (DOC 22 kb)

## Abbreviations

α-MEM: Alpha minimal essential medium
ALP: Alkaline phosphatase
BFR: Bone formation rate
BMD: Bone mineral density
CFA: Complete Freund adjuvant
CHP: Cholesterol-bearing pullulan
CIA: Collagen-induced arthritis
Conn.D: Degree of connectivity of trabeculae normalized by TV
μCT: Microfocal computed tomography
CTX: C-telopeptide fragments of type I collagen
DMARD: Disease-modifying antirheumatic drug
DMSO: Dimethyl sulfoxide
DXA: Dual X-ray absorptiometry
EIA: Enzyme immunoassay
FBS: Fetal bovine serum
Kd: Equilibrium constant
Koff: Binding dissociation constant
MAR: Mineral apposition rate
M-CSF: Macrophage colony-stimulating factor
MMA: Methyl methacrylate monomer
MMP: Matrix metalloproteinase
MS/BS: Mineralizing surface per bone surface
N.Oc/BS: Osteoclast number per bone surface
N.Oc/BV: Osteoclast number per bone volume
OPG: Osteoprotegerin
PBS: Phosphate-buffered saline
pQCT: Peripheral quantitative computed tomography
PTH: Parathyroid hormone
RA: Rheumatoid arthritis
RANK: Receptor activator of nuclear factor-κB
RANKL: receptor activator of nuclear factor-κB ligand
ROI: Region of interest
siRNA: Small interfering RNA
SMI: Structure model index
Tb.N: Trabecular number
TBPf: Trabecular bone pattern factor
TNFα: Tumor necrosis factor alpha
TRAP: Tartrate-resistant acid phosphatase
V m.space: Marrow space star volume
