# Supplementary figures and images for: The inhibitory effects of a RANKL-binding peptide on articular and periarticular bone loss in a murine model of collagen-induced arthritis: a bone histomorphometric study
Source: Arthritis Res Ther. 2015 Sep 12;17(1):251. doi: 10.1186/s13075-015-0753-8 (PMC4570694; doi:10.1186/s13075-015-0753-8)

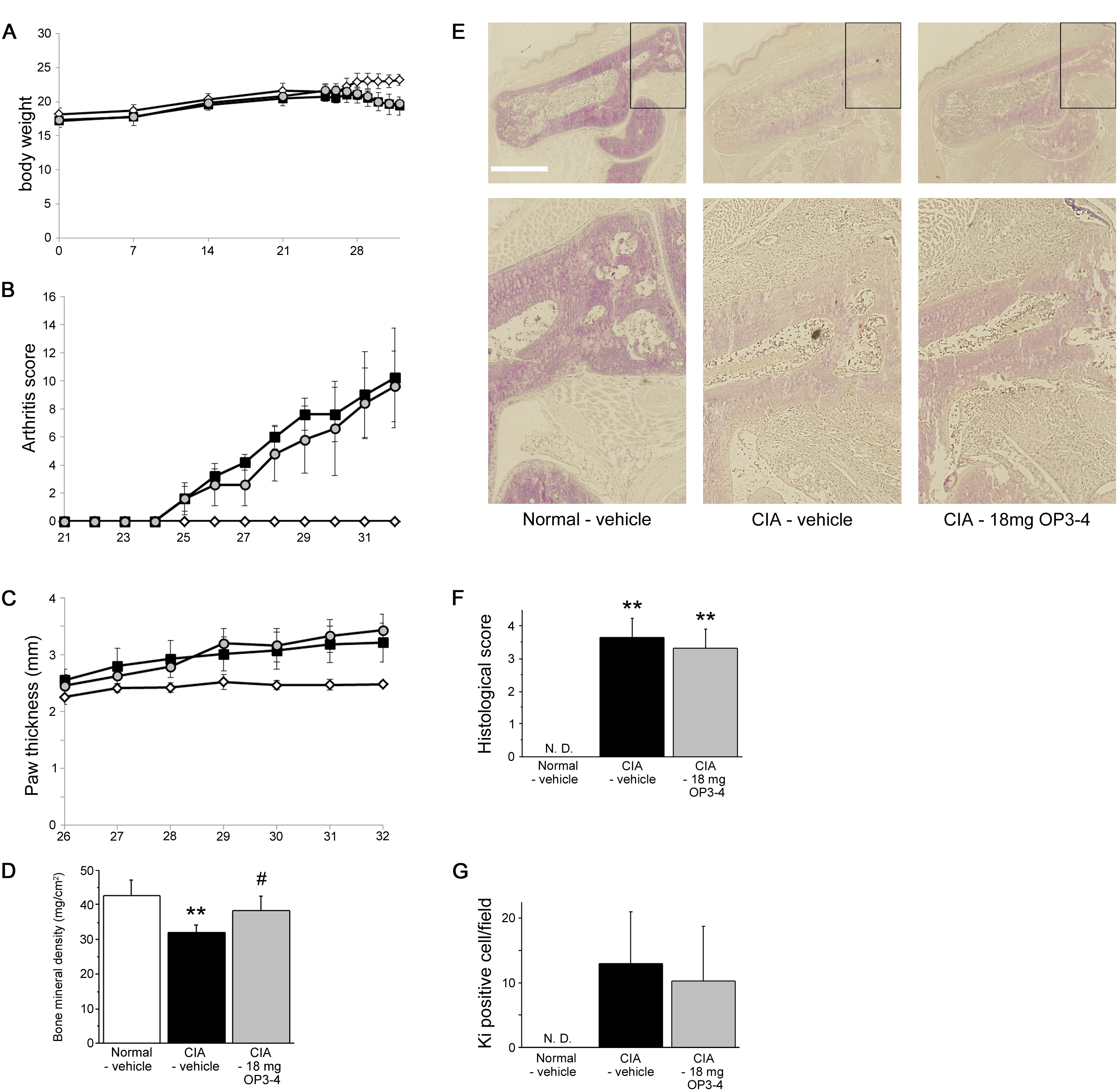

Supplement: Additional file 1: Figure S1. — Showing that OP3-4 administration has a limited effect on the induction and inflammation of arthritis. A–C The osmotic minipumps filled with 20 % DMSO (vehicle) or OP3-4 peptide (to deliver 18 mg/kg/day) were implanted subcutaneously before the first immunization. Body weight A, arthritis score B, and paw swelling by caliper measurement C of mice with CIA treated with vehicle (closed squares), mice with CIA treated with 18 mg/kg/day OP3-4 peptide (closed circles), and normal mice treated with vehicle (open diamond) are indicated. There is no significant difference between vehicle-treated and OP3-4-treated mice. D BMD measured by DXA. **p <0.01 vs. Normal-vehicle, # p <0.05 vs. CIA-vehicle. E Histological images of hematoxylin and eosin staining of hind limb heels are shown. Lower panels, higher magnification of the squares in the upper panels. Bars indicate 1 mm. F Inflammatory scores in each group of mice show no significant difference between CIA-vehicle and CIA-OP3-4 mice. **p <0.01 vs. Normal-vehicle. G Numbers of proliferating cells which are positive for Ki-67 antigen counted in the periarticular region. No significant differences are detected. (JPEG 3012 kb) [file 13075_2015_753_MOESM1_ESM.jpeg]

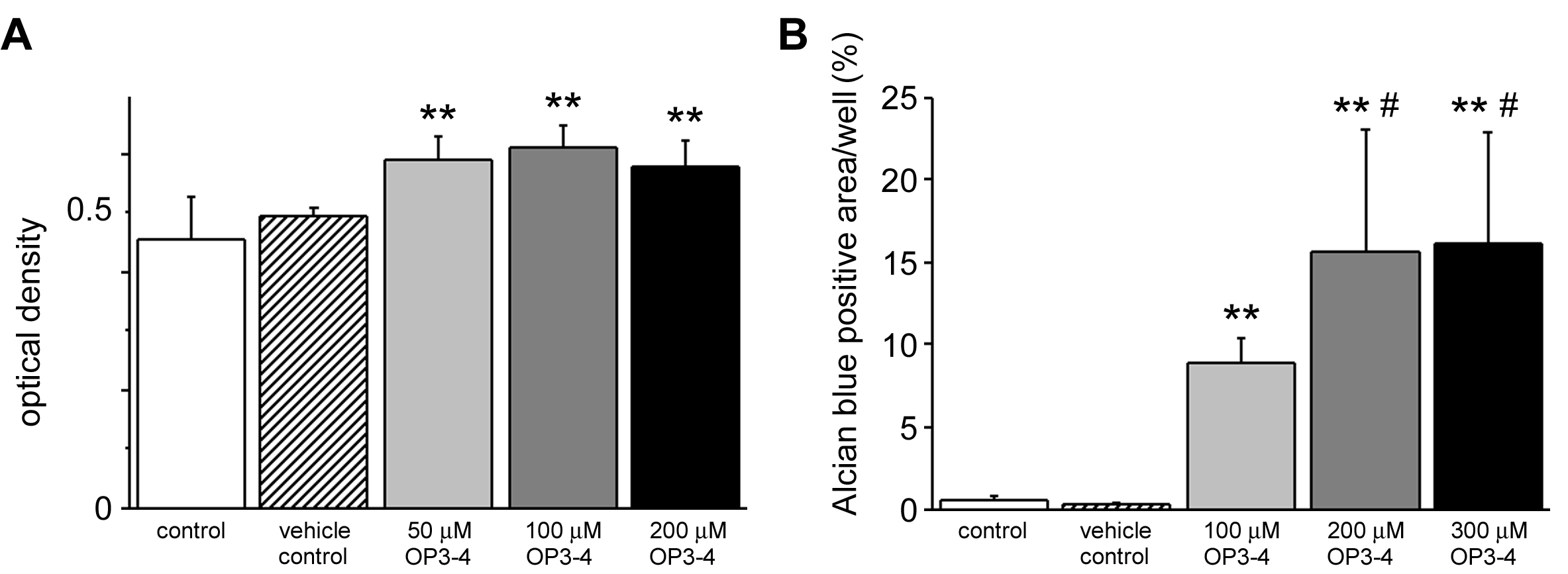

Supplement: Additional file 2: Figure S2. — Showing the effects of OP3-4 on the proliferation and differentiation of cartilage cell line ATDC5. A Results of proliferation assay on day 1 with the noninduction medium. B Alcian blue-positive area ratio in the cartilage induction medium on day 10. **p <0.01 vs. vehicle control, #p <0.05 vs. 100 μM OP3-4. (JPEG 384 kb) [file 13075_2015_753_MOESM2_ESM.jpeg]
