# Supplementary material for: The inhibitory effects of a RANKL-binding peptide on articular and periarticular bone loss in a murine model of collagen-induced arthritis: a bone histomorphometric study
Source: Arthritis Res Ther. 2015 Sep 12;17(1):251. doi: 10.1186/s13075-015-0753-8 (PMC4570694; doi:10.1186/s13075-015-0753-8)
Supplement: Additional file 3: — Presents supplementary methods. (DOC 22 kb) [file 13075_2015_753_MOESM3_ESM.doc]

**Supplementary methods**

Immunohistochemistry

Frozen sections of the heels fixed with 10% formaldehyde were made, and antigen retrieval was performed in 0.01M sodium citrate buffer (pH 6.0) by using microwave oven at 500 W for 3 min. The endogenous peroxidase activity was inactivated by 0.3% H_2_O_2_ in methanol for 30 min. Mouse anti-Ki-67 monoclonal antibody (8D5, Cell signaling Tech., Danvers, MA, USA), SignalStain Boost IHC Detection Reagent (Cell signaling Tech.), and DAB peroxidase substrate (Vector Lab., Burlingame, CA, USA) were used for the detection of Ki-67 positive cells. The numbers of Ki-67 positive cells in the periarticular region were counted.
